# Using the half normal distribution to quantify covariate balance in cluster-randomized pragmatic trials

**DOI:** 10.1186/s13063-021-05122-x

**Published:** 2021-03-06

**Authors:** Jin Huang, David L. Roth

**Affiliations:** Center on Aging and Health, Division of Geriatric Medicine and Gerontology, Johns Hopkins University, 2024 East Monument Street, Baltimore, MD 21205 USA

**Keywords:** Pragmatic trials, Cluster-randomized trials, Re-randomization, Half-normal distribution, Covariate-constrained randomization

## Abstract

**Background:**

Pragmatic trials often consist of cluster-randomized controlled trials (C-RCTs), where staff of existing clinics or sites deliver interventions and randomization occurs at the site level. Covariate-constrained randomization (CCR) methods are often recommended to minimize imbalance on important site characteristics across intervention and control arms because sizable imbalances can occur by chance in simple randomizations when the number of units to be randomized is relatively small. CCR methods involve multiple random assignments initially, an assessment of balance achieved on site-level covariates from each randomization, and the final selection of an allocation that produces acceptable balance. However, no clear consensus exists on how to assess imbalance or identify allocations with sufficient balance. In this article, we describe an overall imbalance index (*I*) that is based on the mean of the absolute value of the standardized differences in means on the site characteristics.

**Methods:**

We derive the theoretical distribution of *I*, then conduct simulation studies to examine its empirical properties under the varying covariate distributions and inter-correlations.

**Results:**

*I* has an expected value of 0.798 and, assuming independent site characteristics, a variance of 0.363/*k*, where *k* is the number of site characteristics being balanced. Simulations indicated that the properties of *I* are robust under varying covariate circumstances as long as *k* is greater than 3 and the covariates are not too highly inter-correlated.

**Conclusions:**

We recommend that values of *I* below the 10th percentile indicate sufficient overall site balance in CCRs. Definitions of acceptable randomizations might also include individual covariate criteria specified in advance, in addition to overall balance criteria.

Pragmatic trials are increasingly proposed as a way to implement and evaluate new intervention or treatment programs directly into clinical practice settings [1, 2]. Many interventions, particularly for older adults and their family members, involve multiple intervention components that are delivered by existing staff persons at clinics or other facilities (e.g., [3, 4]). Consequently, cluster-randomized controlled trials (C-RCTs)—where structural units or sites such as clinics, hospitals, community centers, or residential facilities are randomized to intervention or control conditions—are becoming increasingly popular to test the efficacy or effectiveness of these interventions. Multiple individual patients, clients, or residents from each site typically serve as participants and provide person-level outcome data [5, 6].

In many C-RCTs, the number of sites to be randomized can be relatively small. A simple randomization of experimental units to two or more intervention conditions is assured of achieving relative balance across conditions on measured and unmeasured potential confounding variables only if the number of units to be randomized is relatively large. When the number of clusters or sites to be randomized is relatively small, problematic imbalances on potentially important and confounding site characteristics are likely to arise purely by chance. Optimized randomization procedures are often recommended to ensure balance on cluster characteristics across treatment conditions in C-RCTs [6]. Pragmatic trails that use C-RCT methods could be strengthened and made more comparable methodologically by the adoption of rigorous and more uniform procedures in the randomization process.

Optimized randomization procedures for achieving site balance in C-RCTs have been categorized into four general types [7]. These include stratification, matching, minimization, and covariate-constrained randomization (CCR). Ivers and colleagues [7] provided detailed descriptions of these types of procedures, including the strengths and limitations of each approach. Of the four types, CCR is becoming increasingly popular as a method that has numerous advantages, but questions remain about its implementation and the optimal metrics for assessing the balance achieved [8–10]. All CCR approaches share the following features: (1) data on key site characteristics are available to investigators before randomization occurs; (2) multiple random assignments of sites to the treatment arms are conducted before the trial begins, sometimes referred to as re-randomization [11]; (3) the balance achieved on the set of site characteristics is examined for each randomization; and (4) a random assignment to be used in the trial is selected, usually randomly, from among the multiple randomizations deemed to be acceptable.

Moulton [12] developed a classic CCR approach that consisted of specifying, in advance, the minimal degree of imbalance desired on each specific cluster characteristic, conducting all possible randomizations, identifying the subset of possible randomizations that meet balance criteria, and randomly selecting one random allocation from that subset of acceptable randomizations. However, as the number of sites increases, the number of total possible randomizations expands rapidly, and questions remain on how “acceptable balance” should be defined. Ciolino and colleagues [10] endorsed a threshold of acceptable balance that required all *p* values from nonparametric analyses of individual site characteristics by assigned treatment condition to be 0.30 or higher. Although this recommendation ensures that a retained randomization will not have any significant or marginally significant imbalances on any site characteristic, *p* values are somewhat dependent on the number of sites being randomized, and notable but non-significant imbalances may still remain if the number of sites being randomized is relatively small. In addition, as the number of balancing site variables increases, a smaller and smaller proportion of all randomizations will be identified as satisfactory using this criterion.

In addition to defining balance based on individual site characteristic criteria, overall balance across all site characteristics collectively can be considered. Raab and Butcher [8] proposed an overall balance metric that consisted of the weighted sum of squared mean differences across site characteristics, and Li and colleagues [9] described a similar overall imbalance index based on a weighted sum of absolute mean differences across site characteristics. Thresholds of acceptable overall site balance sometimes include recommendations to only include those randomizations whose overall imbalance metrics are in the lower 10% of their respective empirical distributions [8, 9].

In this paper, we develop and describe an overall imbalance index that is based on the absolute value of standardized mean differences calculated from multiple site variables. We point out that the frequency distribution of this imbalance metric is based on the folded normal or half-normal distribution [13, 14]. We examine the statistical properties of our imbalance (*I*) index and compare it to Raab and Butcher’s [8] *B* index as an overall site balance index for use in future C-RCTs with two treatment conditions.

## Calculation of the overall metric of site characteristic imbalance (*I*)

The following procedures are used to determine *I*:

1. For each site characteristic for which balance is desired, calculate the observed difference in means between treatment arms (e.g., intervention − control) after each random allocation to those conditions.
2. Calculate the standard deviation of the mean difference between treatment arms, then divide the observed mean differences by their respective standard deviations to standardize those mean differences.
3. Take the absolute value of each of those standardized mean differences.
4. Calculate the mean of those absolute values from step 3 across the multiple site balancing variables. This mean is the *I* index.

For one site variable and one simple random assignment to two conditions, the expected value under the null hypothesis of a raw mean difference between the two treatment arms is 0. However, the absolute value of this mean difference will not have an expected value of 0. When the absolute value of a normally distributed variable with a mean of 0 is taken, the normal distribution is folded at that population mean (*μ* = 0) and becomes the positively-skewed, half-normal distribution [13, 14]. The mean of the *absolute value of this difference in means* (AVDM) is:

$E\left(\mathrm{AVDM}\right)={S}_{\mathrm{DM}}\ast \sqrt{2/\pi }={S}_{\mathrm{DM}}\ast 0.798$, where ${S}_{DM}\ \left(\mathrm{standard}\ \mathrm{deviation}\ \mathrm{of}\ \mathrm{the}\ \mathrm{difference}\ \mathrm{in}\ \mathrm{means}\right)=\sqrt{S_1^2/{n}_1+{S}_2^2/{n}_2}$, and *S*_j_ is the standard deviation of the raw variable in treatment arm j. Furthermore, the variance of AVDM is given by:

$$ \mathrm{Var}\ \left(\mathrm{AVDM}\right)={S}_{\mathrm{DM}}^2\ast \left(1-2/\pi \right)={S}_{\mathrm{DM}}^2\ast 0.363. $$

For a standardized difference in means, where *S*_DM_ is scaled to be equal 1.0, the expected value and variance of AVDM become 0.798 and 0.363, respectively, and the standard deviation of AVDM = $\sqrt{.363}$ = .602. Because this half-normal distribution for AVDM is positively skewed, the expected values for the mean and median are not equal, and the expected value for the median is 0.674.

When calculating *I*, the standardized AVDMs are averaged across multiple balancing variables (*k*), and the central limit theorem applies to the distribution of this average. This results in a distribution that approximates the normal distribution as *k* becomes sufficiently large. As *k* increases, the median approaches the mean of 0.798, and if the covariates are assumed to be independent, then the variance of *I* becomes 0.363/*k* (SD = $0.602/\sqrt{k}$). Thus, the imbalance metric *I* should approximate 0.798 for any single randomization, and the extent to which it is less than 0.798 will reflect the degree of reduced imbalance achieved by that particular random allocation compared to what would be expected by chance. Once the number of balancing variables (*k*) is known, the degree of overall imbalance can be calculated using either standard deviation units or percentile rankings.

In order to examine the statistical properties of *I* in a standard 2-arm C-RCT, we conducted multiple simulation studies under a variety of conditions that might occur in practice. Although there is no gold standard to evaluate overall covariate imbalance, we compared the *I* index with the *B* index of Raab and Butcher [8] and with the acceptability decisions that would have been provided by the criteria of Ciolino and colleagues [10]. We simulated cluster or site-level variables, where the variables were drawn from normal, Bernoulli, or lognormal distributions, and with different levels of inter-correlation for the multivariate normal simulations. Under each distribution and inter-correlation combination, we examined simulated results when 12, 18, or 60 sites were randomized and whether randomization was done in a 1:1 or 1:2 allocation ratio. These simulations allowed us to examine the robustness of *I* and its distributional properties under multiple varying conditions.

## Methods

Simulation 1 assessed the effect of varying number of site balancing variables and correlation among these variables on the statistical properties of the imbalance (*I*) index. We assumed that the site balancing variables come from a multivariate normal distribution. We varied the number of variables from 2 to 4 and examined a range of 4 equally spaced correlations from 0 to 0.75 among these variables (i.e., 12 scenarios). Descriptive statistics of *I* were computed from the simulated data and compared with statistics theoretically derived based on central limit theorem and with the simulated *B* index.

Simulation 2 examined the dependence of the *I* index on the distributions and skewness of the site-level variables. In addition to the normal distribution, Bernoulli and lognormal distribution are commonly used non-normal distributions in health care research. We generated the following data:

1. *X*_1_, *X*_2_, *X*_3_, *X*_4_ ~ MVN(*μ*, Σ), where *μ* = (0, 0, 0, 0)’ and *Σ* = I
2. *X*_1_ ~ Bernoulli (0.3), *X*_2_ ~ Bernoulli (0.5), *X*_3_, *X*_4_ ~ Normal (0, 1)
3. *X*_1_ ~ lognormal (0, 1), *X*_2_ ~ lognormal (0, 1), *X*_3_, *X*_4_ ~ Normal (0, 1)

We assumed the variables were drawn independently in each of these scenarios.

The simulation studies focused on two treatment arms. To evaluate the dependence on the number of sites, we considered 12, 18, or 60 sites. Besides the 1:1 design, which is most commonly implemented in C-RCTs, we also examined a 1:2 allocation ratio as well. For 18 and 60 sites, we simulated 10,000 unique randomizations without duplicate by assigning the 18 or 60 sites into two treatment arms with 1:1 or 1:2 allocation ratios. For the 1:1 allocation ratio and 18 sites, there are 24,310 unique distinct splits of 9 vs. 9 sites, and there are over 1,000,000 distinct randomizations when the number of sites is 24 or larger. Earlier simulations indicated that 10,000 unique randomizations were sufficient for examining the statistical properties and distributions of the *I* index and that it was not necessary to conduct all possible randomizations. For 12 sites, we conducted all possible randomizations into the two treatment arms, which involved 462 randomizations for the 1:1 allocation ratio and 495 for the 1:2 allocation ratio. Random assignments for each site were determined by random numbers generated by the SAS statistical software package (SAS 9.4, Cary NC).

For each randomization, we calculated the overall imbalance metrics (*I* and *B*) and examined individual site characteristic *p* values for differences by the two groups using the Kruskal-Wallis (KW) statistic as recommended by Ciolino and colleagues [10]. The randomizations were identified as sufficiently balanced if the pre-specified balancing criteria were met. For *I* and *B*, we chose the lowest 10% to signify sufficient balance. For KW, we specified a min (*P* value) > 0.30 as the threshold of sufficient balance [10].

### Calculation of the overall balance metrics

We calculated the *B* index, which is a sum of weighted squared differences across site-level variables, using the method described by Raab and Butcher [8], namely:

$$ B=\sum \limits_{i=1}^k{w}_i{\left({\overline{x}}_{0i}-{\overline{x}}_{1i}\right)}^2 $$

where *k* denotes the number of site-level variables to be balanced, 2 to 4 in our simulations; *w*_*i*_ is a pre-specified weight for the *i*th site-level variable; and ${\overline{x}}_{0i}$, ${\overline{x}}_{1i}$ are the means of site-level variable for the two treatment arms. Consistent with guidance from Raab and Butcher [8], we used the inverse of the variance of the difference in means for each variable as its weight (*w*_*i*_). This results in each site variable being equally weighted in the calculation of *B*. If the *k* site-level variables are independent of each other, then this weighting results in *B* being distributed as a chi-square statistic with *k* degrees of freedom.

For *I*, we first calculated absolute values of standardized mean differences for each site characteristic and then averaged across multiple balancing variables.

$$ I=\frac{1}{k}\sum \limits_{i=1}^k\frac{1}{S_{DMi}}\mid {\overline{x}}_{0i}-{\overline{x}}_{1i}\mid $$

where *S*_*DMi*_ is the standard deviation of the difference in means for the *i*th site-level variable. As stated previously, if the *k* site-level variables are assumed to be independent of each other, then *I* ~ *N* (0.798, $0.602/\sqrt{k}\Big).$

The *B* and *I* indices, therefore, are both designed to assess overall imbalance across multiple site-level variables and are conceptually somewhat similar. The *I* index relies on absolute values of standardized mean differences and approximates the normal distribution regardless of the number of site characteristics, and the *B* index relies on squared mean differences and can be weighted to approximate the chi-square distribution.

## Results

Across all simulations, the differences by allocation ratio (1:1 vs. 1:2) were minimal. Thus, because the 1:1 allocation ratio is the most commonly used approach in practice, only the results from that allocation ratio are further presented here.

**Fig. 1:**
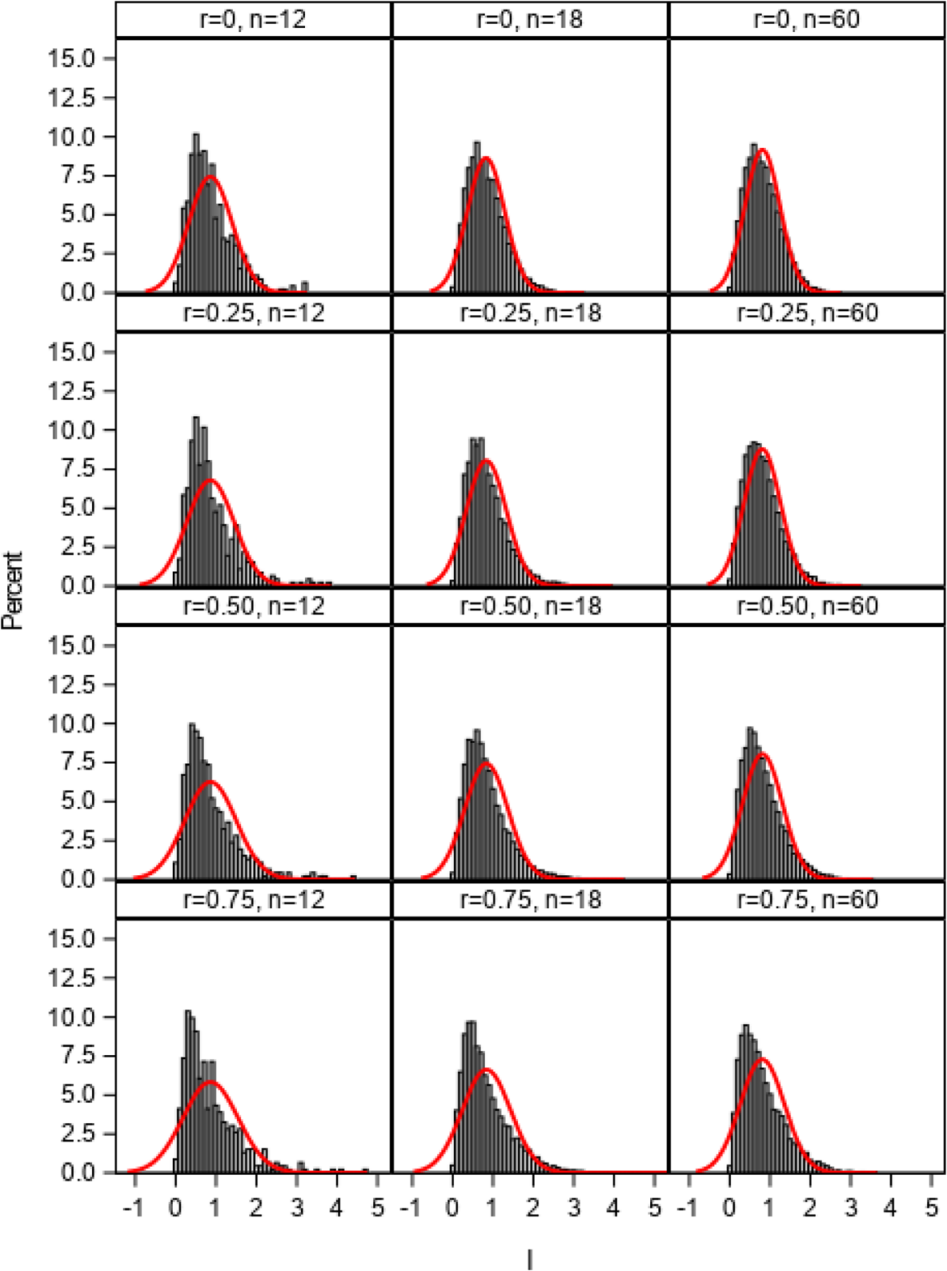
Distributions of the imbalance (*I*) index and estimated normal density curves with 2 cluster-level variables

**Fig. 2:**
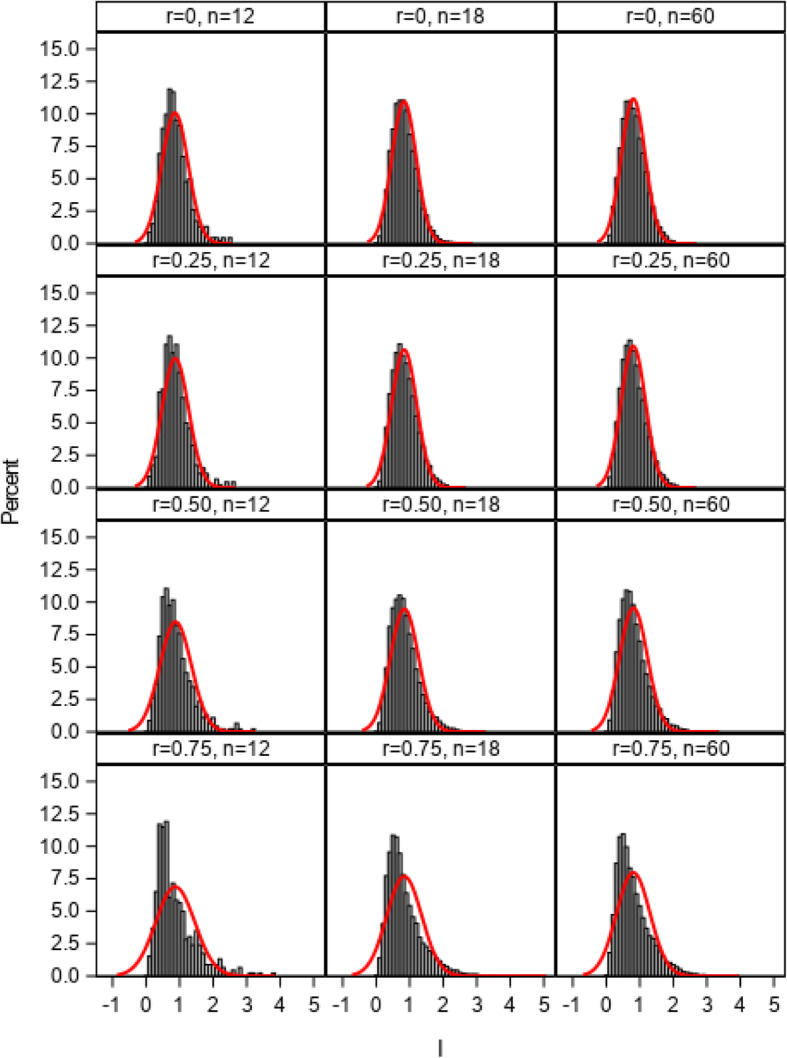
Distributions of the imbalance (*I*) index and estimated normal density curves with 3 cluster-level variables

**Fig. 3:**
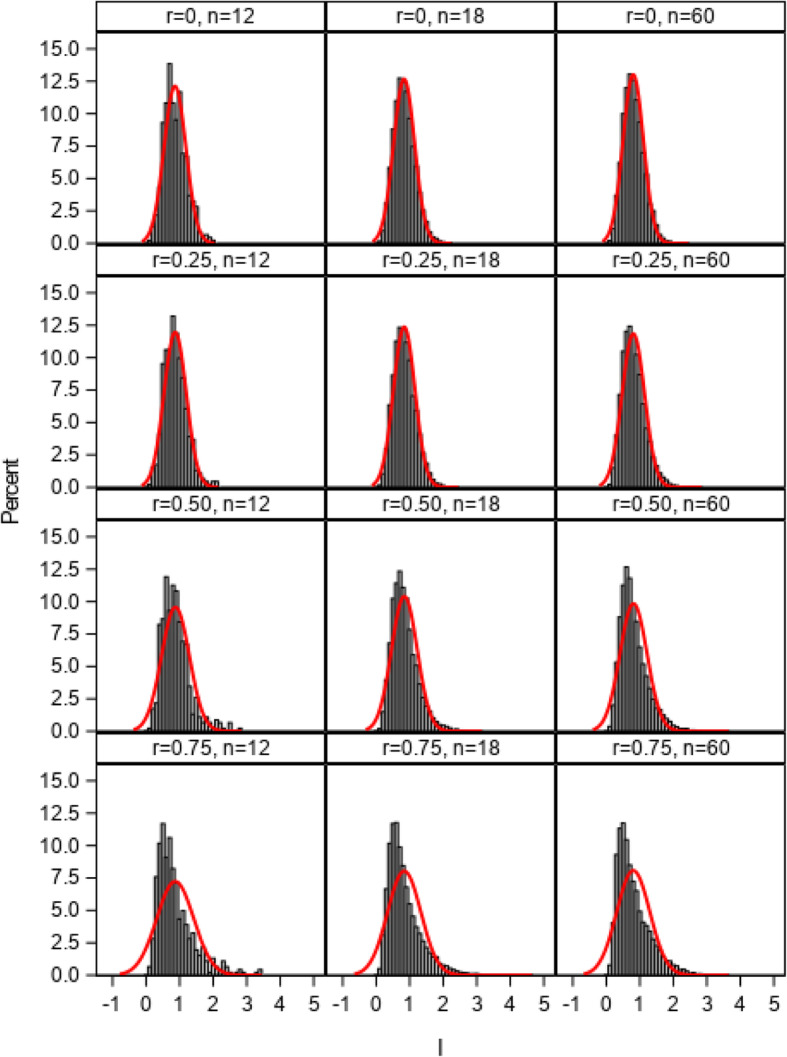
Distributions of the imbalance (*I*) index and estimated normal density curves with 4 cluster-level variables

The distributions of the *I* index from 1:1 allocation ratio simulations using 2, 3, and 4 multivariate normal distributed cluster-level variables with correlations ranging from 0 to 0.75 (simulation 1) are illustrated in Figs. 1, 2, and 3. The estimated normal density curves are overlaid. The observed distributions were found to closely approximate the normal distribution when correlations among the site variables were low (0.25 or less) with three or more cluster-level variables across the 12-, 18-, and 60-site simulations. For more moderately correlated (*r* = 0.50) or highly correlated (*r* = 0.75) site variables, the observed distributions tended to more closely approximate the normal distribution as the number of simulation sites increased to 60 and as the number of site variables increased to 4, but some moderate deviations from normality were still evident. The more extreme the correlation, the greater the deviation of the distribution from normal distribution due to the violation of independent covariate assumption in central limit theorem. Because the empirical I index could never be less than 0, deviations between the observed distributions and the overlaid normal density curves tended to be greatest in the lower tails of those distributions.

**Fig. 4:**
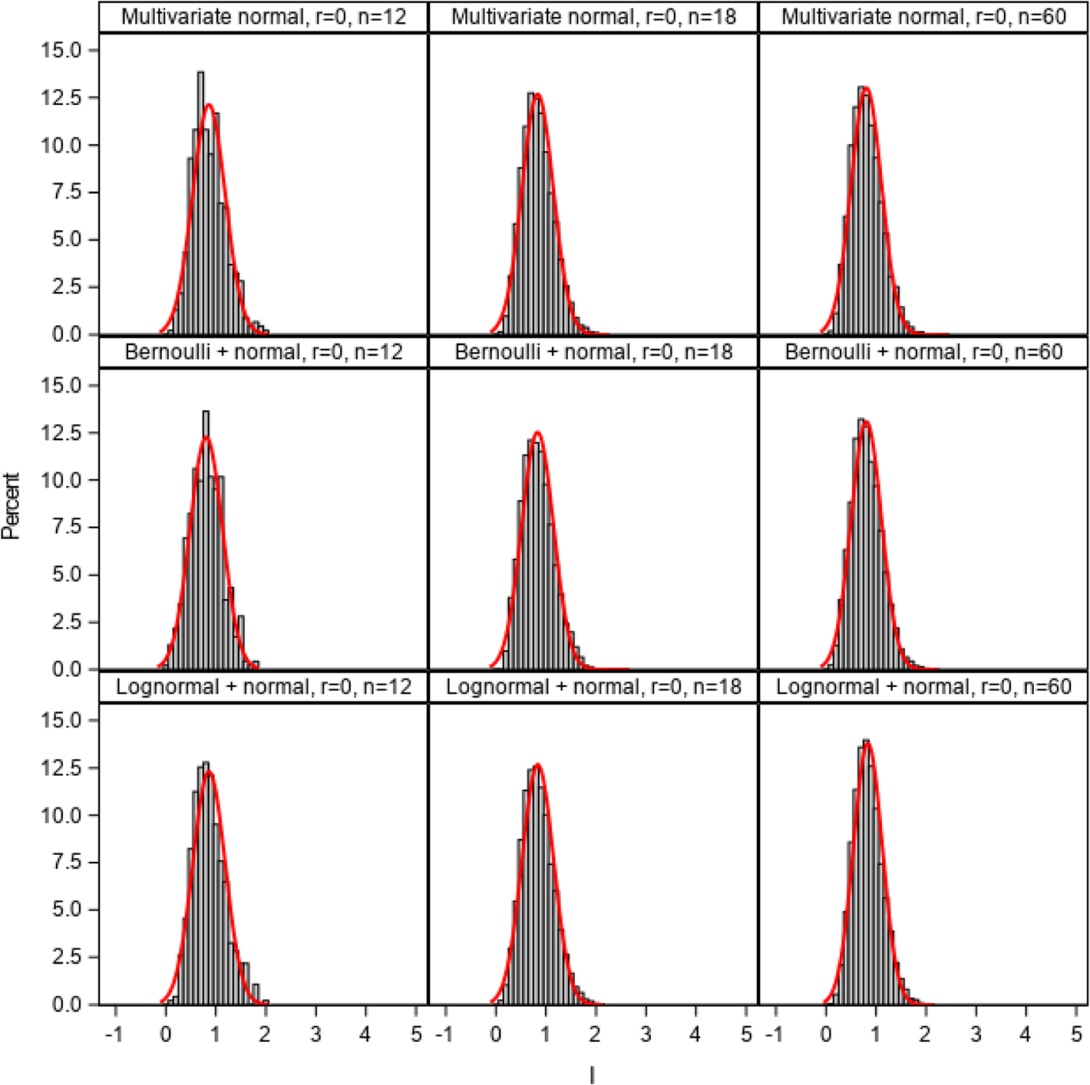
Distributions of the imbalance (*I*) index and estimated normal density curves for cluster-level variables with various distributions

Figure 4 illustrates the distributions of the *I* index from simulations that included 4 uncorrelated site-level variables with different combinations of normal, Bernoulli, and lognormal distributions (simulation 2). There were minimal differences in the distribution of the I index regardless of the distributions of the site-level variables, suggesting that the *I* index of imbalance can be confidently used with a mix of different variable types as long as those site variables are minimally correlated.

**Fig. 5:**
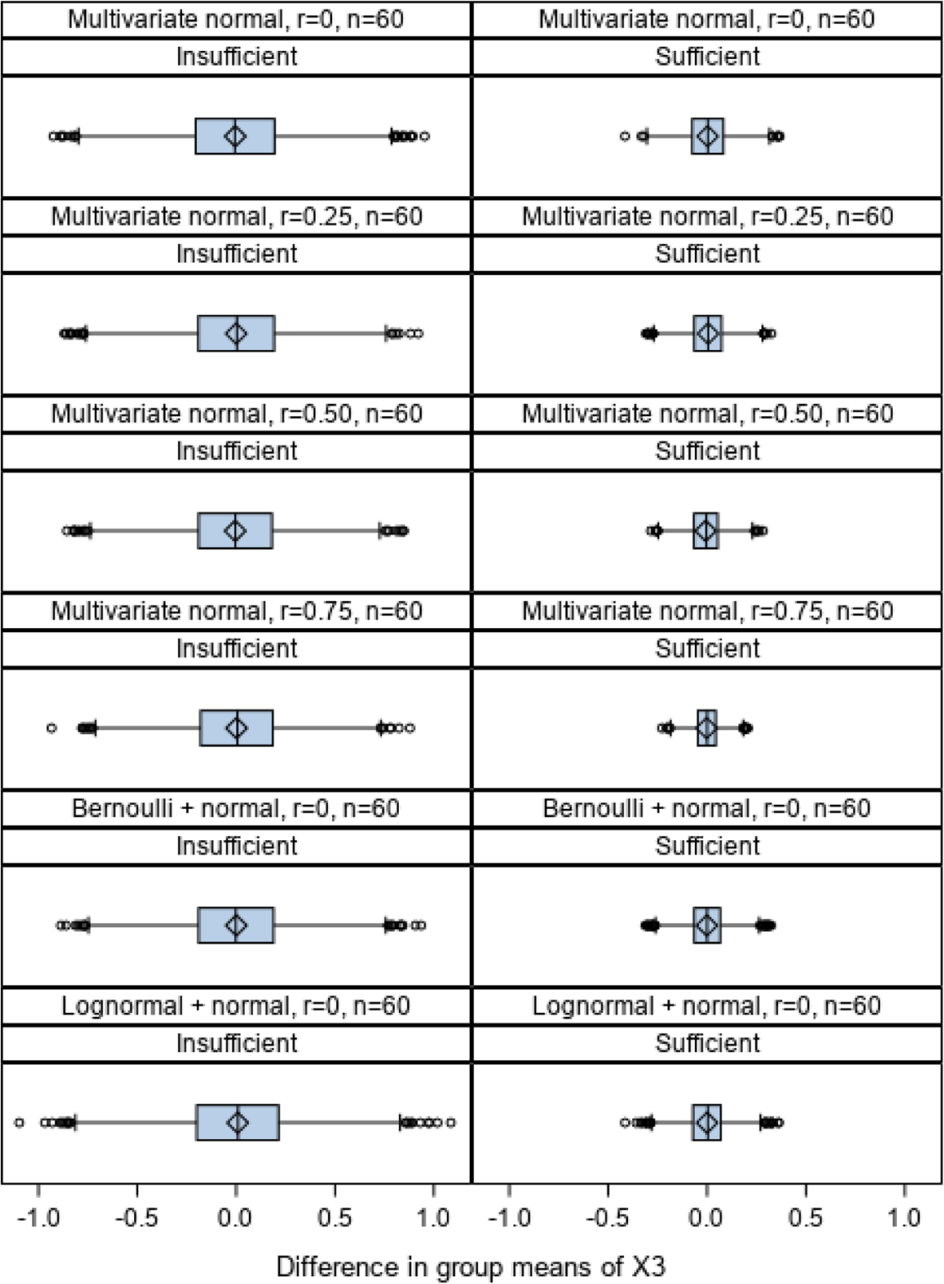
Distributions of group difference over 10,000 randomizations for sufficiently and insufficiently balanced randomizations

We anticipate that the mean difference of site-level variables between two arms equals zero, while the standard deviation of the group mean difference for allocations deemed as sufficiently balanced should be markedly reduced compared with insufficiently balanced allocations. To examine this, we selected one site-level variable (X_3_) from the four variable simulations and examined the distributions of group differences of X_3_ separately for sufficiently and insufficiently balanced allocations as determined by the 10th percentile of the *I* index. These results are displayed in Fig. 5. For each scenario, sufficiently balanced randomizations demonstrated much smaller variations in differences by assigned treatment condition.

Tables 1 and 2 contain comparative information from uncorrelated multivariate normal simulations for the overall balance indices (*I* and *B*) and for the criterion that each individual characteristic have a KW *p* value > 0.30. As the number of site variables increased, the mean for *I* stays relatively constant and the SD decreases per the central limit theorem, whereas both the mean and SD of *B* increase, consistent with the increasing degrees of freedom of the chi-square distribution. Additional analyses examined the associations between *I* and *B* from the multivariate normally distributed variables. The two metrics were closely correlated with Spearman correlation coefficients ranging from 0.96 to 0.99. Across all simulated scenarios, *I* and *B* were in agreement for over 96% of the simulations in terms of whether those indices were in the bottom 10% of their respective distributions, with kappa agreement statistics ranging from 0.84 to 0.91. For the KW *p* value criterion, the proportion of acceptable randomizations is much larger when the number of site characteristics is small but steadily decreases as the number of site characteristics increases. Kappa statistics of agreement between the KW *p* value criterion and both *I* and *B* were small to medium in magnitude, ranging from 0.21 with both *I* and *B* when only 2 site characteristics were examined and increasing steadily as the number of site characteristics increased. Kappa was 0.44 in relation to *I* and 0.47 in relation to *B* for 4 uncorrelated, multivariate normal site characteristics.

**Table 1:** Properties of *I* and *B* based on simulations of uncorrelated multivariate normal distributed variables using the 1:1 allocation ratio for 60 sites

# of site-level variables	Mean	*I*			Mean	*B*		
		SD	10th percentile	25th percentile		SD	10th percentile	25th percentile
2	0.815	0.435	0.291	0.486	2.100	2.109	0.210	0.598
3	0.809	0.357	0.372	0.545	3.114	2.580	0.573	1.216
4	0.807	0.307	0.434	0.584	4.125	2.961	1.082	1.955

*SD* standard deviation

**Table 2:** Number of randomizations that met pre-specified balance criteria from 10,000 randomizations of uncorrelated multivariate normal distributed variables using the 1:1 allocation ratio for 60 sites

# of site-level variables	*I*	*B*	All KW *p* values > 0.30
2	1000	1000	4757
3	1000	1000	3433
4	1000	1000	2388

*KW* Kruskal-Wallis

Table 3 provides descriptive information from theoretical normal distributions of *I* for up to 10 independent site characteristics. Theoretical percentile cutpoints are provided for thresholds of both highly balanced (10th percentile) and moderately balanced (25th percentile) randomizations. The theoretical percentile cutpoints for 2, 3, and 4 site variables in Table 3 can be compared to the empirical cutpoints from the 60-site simulations reported in Table 1. The theoretical 10th percentile values are slightly lower than the empirical values, whereas the theoretical 25th percentile values are slightly higher. Additional analyses examined the concordance of acceptable balance based on 10th percentile for theoretical and empirical cutpoints. In over 98% of randomizations, the same acceptability decision was made. Because the theoretical 10th percentile cutpoints were slightly lower, discordant decisions occurred when the observed *I* index was acceptable in comparison to the empirical thresholds but not acceptable in relation to the theoretical cutpoint. The 10th percentile theoretical cutpoints in Table 3, therefore, are slightly conservative and may identify as acceptable slightly less than 10% of the actual randomizations.

**Table 3:** Properties of *I* based on the normal distribution results from the central limit theorem applied to the half-normal distribution for 1 to 10 site-level variables

# of site-level variables	Mean	SD	10th percentile	25th percentile
1^a^	0.798	0.602	0.026	0.392
2	0.798	0.426	0.252	0.511
3	0.798	0.348	0.352	0.563
4	0.798	0.301	0.412	0.595
5	0.798	0.269	0.453	0.616
6	0.798	0.246	0.483	0.632
7	0.798	0.228	0.506	0.644
8	0.798	0.213	0.525	0.654
9	0.798	0.201	0.541	0.663
10	0.798	0.191	0.554	0.669

*SD* standard deviation

^a^Distribution is clearly not normal with only 1 site variable, so theoretical percentile values may be misleading in this case

## Discussion

This paper builds on previous efforts [8, 9] to develop and apply overall balance criteria that aggregate information across multiple covariate characteristics from randomized controlled trials. Such methods are often helpful for balancing clusters or sites across treatment conditions in C-CRTs, although these methods can be used in other types of randomized trials as long as the units to be randomized and their characteristics are known ahead of time. We showed that the average of standardized absolute mean differences between two arms (e.g., treatment vs. control) across multiple site characteristics is an index that is relatively normally distributed with a mean of 0.798 and a variance of 0.363/k as long as k, the number of site characteristics being balanced, is sufficiently large and the correlations among the site characteristics are minimal.

The *I* index is an advance over previous work in that it provides a standardized metric that can be used for multiple purposes. Like the *B* index described by Raab and Butcher [8], the *I* index can be used to guide the selection of random allocations in C-CRTs that demonstrate sufficient overall balance on a set of covariates or site characteristics collectively. Due to its standardization and the distributional properties illustrated here, the *I* index also provides a metric of the overall degree of imbalance across all site characteristics that can be interpreted, at least approximately, into percentile ranks from the theoretical normal distribution. Percentile ranks can also be obtained from the chi-square distribution for the *B* index and used similarly. The metrics could be used to conduct comparisons of the level of imbalance obtained in other trials that might use a different number of sites or site characteristics. Both *I* and *B* can be calculated for quantitative or categorical variables; for categorical variables with j different categories, j – 1 dummy-coded (1 vs. 0) variables can be created and the method otherwise proceeds as outlined here.

Our simulations generally indicate that 3 or more minimally or only moderately correlated site characteristics are sufficient to assume normality in most cases, especially if 18 or more sites are available to be randomized. The theoretical percentile ranks and cutpoints should be particularly useful to investigators in these cases because they allow an estimation the degree of imbalance in a particular randomization without conducting all possible randomizations and calculating empirical cutpoints. As the number of sites increases, it becomes unnecessary and perhaps even cumbersome to conduct all possible randomizations, which quickly can expand to hundreds of thousands or even millions of distinct random allocations. Using the *I* index, a few hundred randomizations will typically be adequate to identify a sufficient number of acceptable randomization allocations from which one might be randomly selected for implementation in the trial. In cases where the number of sites is relatively small (e.g., < 18) or the correlations among some site characteristics are high (e.g., > 0.50), the theoretical cutpoints from the normal approximation may be misleading. We recommend that empirical percentile cutpoints using either *I* for *B* from a sufficiently large number of randomizations be used to identify suitable randomizations in these situations.

Limitations to our work include the many restrictions we implemented in our simulations. We recognize that only a small and finite number of variable conditions were simulated, including no more than 4 site characteristics and only two treatment conditions. We are confident that the distributions of *I* would continue to even more closely approximate the normal distribution as the number of site characteristics increases or as the number of sites to be randomized increases. Future simulations might examine modifications to the *I* index when randomizations to more than two treatment conditions are desired. Future work is also needed to examine additional mixtures of site characteristic distributions and inter-correlation patterns.

Another important area where future research is needed is on the implications of optimized randomization procedures for the statistical analysis of treatment effects on outcomes. In most cases, the increased balance achieved on covariates from optimized randomization procedures is expected to result in more precise estimates of treatment effects and greater power, potentially rendering traditional parametric analyses overly conservative [11, 15]. Because the *I* index is a standardized, comparative metric of reduced imbalance, it may be useful as a metric for future simulation studies that examine associations between the degree of balance achieved and the alterations in observed type I or type II error rates.

As pragmatic trials continue to evolve and become more dominant sources of evidence for both the efficacy and effectiveness of interventions [16], it will be important for methodological and research design innovations to keep up with the increased complexity that is often encountered in such investigations. Pragmatic trials that use C-CRT designs should generally not rely on simple randomizations or limited approaches such as stratified random assignment [7]. While seeking greater innovation, it will be important for investigators using C-CRTs to exercise caution and ensure that treatment allocation decisions are not impacted improperly by specific site preferences or other interim decisions that might introduce an appearance of bias [12]. Any time multiple possible randomizations are conducted and reviewed, sources of bias might creep in if sufficient safeguards are not in place.

We recommend that randomization method decisions and balance acceptability thresholds be established before individual allocations to treatment conditions are reviewed. Moulton [12] has previously discussed the risks of tightening or relaxing study-specific covariate criteria at interim stages of a trial and stated that these and other overly constraining manipulations can convey impressions that investigators have “rigged the outcome (p. 301)” or “manipulated the design to his or her advantage. (p. 304).” It is important, therefore, to establish such procedures and decision thresholds before the final allocation is determined and treatment assignment information is communicated to the sites. Typically, this means that randomization procedures are executed before the trial begins by statisticians or research design experts who have no direct contact with project sites or their personnel. In some cases, an overall balance criterion, such as the 10th percentile or the 25th percentile of the theoretical distribution for *I* as provided in Table 3, will be sufficient. In other studies, an overall balance criterion might be used in conjunction with specific criteria for one or more individual site characteristics, such as some minimum *p* value for all statistical comparisons of those individual site characteristics. In general, as investigators hope to optimize research designs and maintain rigor in their pragmatic trials, making these decisions carefully before the trial is initiated should ensure sufficient balance across treatment conditions and eliminate any opportunities for biased treatment assignments.

## Data Availability

These simulated data and the software codes that generated and analyzed the data will be made available by the authors upon written request.

## References

[1] Ford I, Norrie J (2016). The changing face of clinical trials: pragmatic trials. N Engl J Med.

[2] Thorpe KE, Zwarenstein M, Oxman AD (2009). A pragmatic-explanatory continuum indicator summary (PRECIS): a tool to help trial designers. J Clin Epidemiol.

[3] Bhasin S, Gill TM, Reuben DB, Latham KK, Gurwitz JH, Dykes P (2018). Strategies to reduce injuries and develop confidence in elders (STRIDE): a cluster-randomized pragmatic trial of a multifactorial fall injury prevention strategy: design and methods. J Gerontol A Biol Sci Med Sci.

[4] Gitlin LN, Marx K, Scerpella D, Dabelko-Schoeny H, Anderson KA, Huang J (2019). Embedding caregiver support in community-based services for older adults: a multi-site randomized trial to test the Adult Day Service Plus Program (ADS Plus). Contemp Clin Trials.

[5] Donner A, Klar N (2000). Design and analysis of cluster randomization trials in health research.

[6] Hayes RJ, Moulton LH (2017). Cluster randomised trials.

[7] Ivers NM, Halperin IJ, Barnsley J, Grimshaw JM, Shah BR, Tu K (2012). Allocation techniques for balance at baseline in cluster randomized trials: a methodological review. Trials..

[8] Raab GM, Butcher I (2001). Balance in cluster randomized trials. Stat Med.

[9] Li F, Turner EL, Heagerty PJ, Murray DM, Vollmer WM, DeLong ER (2017). An evaluation of constrained randomization for the design and analysis of group-randomized trials with binary outcomes. Stat Med.

[10] Ciolino JD, Diebold A, Jensen JK, Rouleau GW, Koloms KK, Tandon D (2019). Choosing an imbalance metric for covariate-constrained randomization in multiple-arm cluster-randomized trials. Trials..

[11] Morgan KL, Rubin DB. Rerandomization to improve covariate balance in experiments. Ann Stat. 2012;40(2):1263–82.

[12] Moulton LH (2004). Covariate-based constrained randomization of group-randomized trials. Clin Trials.

[13] Leone FC, Nelson LS, Nottingham RB (1961). The folded normal distribution. Technometrics..

[14] Tsagris M, Beneki C, Hassani H (2014). On the folded normal distribution. Mathematics..

[15] Li F, Lokhnygina Y, Murray DM, Heagerty PJ, DeLong ER (2016). An evaluation of constrained randomization for the design and analysis of group-randomized trials. Stat Med.

[16] Curran GM, Bauer M, Mittman B, Pyne JM, Stetler C (2012). Effectiveness-implementation hybrid designs: combining elements of clinical effectiveness and implementation research to enhance public health impact. Med Care.

